# Supplementary material for: Self-regulating and self-oscillating metal-organic framework hybrid plasmonic metasurfaces
Source: Nat Commun. 2025 Nov 24;16:10392. doi: 10.1038/s41467-025-65338-2 (PMC12644829; doi:10.1038/s41467-025-65338-2)
Supplement: Supplementary file 1 — Supplementary Information [file 41467_2025_65338_MOESM1_ESM.pdf]

Supplementary Information

# Self-regulating and self-oscillating Metal-Organic Framework based plasmonic metasurfaces

Hajar Amyar,[1,2] Davide Raffaele Ceratti,[1] Henri Benisty,[2] Andrea Cattoni,[3] Mondher Besbes,[2] Marco Faustini[1,4]*

1 Sorbonne Université, CNRS, Laboratoire Chimie de la Matière Condensée de Paris (LCMCP), F-75005 Paris, France

2 Université Paris-Saclay, Institut d'Optique Graduate School, CNRS, Laboratoire Charles Fabry, 91127 Palaiseau, France

3 Centre de Nanosciences et de Nanotechnologies (C2N), CNRS UMR 9001, Université Paris-Saclay, Palaiseau, France.

4 Institut Universitaire de France (IUF), 75231 Paris, France

E-mail: marco.faustini@sorbonne-universite.fr

**Supplementary Table 1**

**State of the art on MOF hybrid plasmonic materials**

In recent years, MOF hybrid plasmonic materials have emerged as multifunctional platforms combining the sorption preperties of MOFs with the field-enhancing capabilities of metallic nanostructures. These hybrid architectures have shown promise in various domains, including photocatalysis, drug delivery, and ultra-sensitive molecular sensing (e.g., SERS). Their performance depends critically on the nanostructural design, including core–shell geometries, interface engineering, and the type of MOF and plasmonic metal used.

The table below illustrates several key examples from the literature that highlight the diversity of MOF hybrid plasmonic nanostructures and their applications in sensing, catalysis, and therapy:

| Publication | Type of MOF | Plasmonic Nanoparticles | Structure | Applications |
|---|---|---|---|---|
| Dhakshinamoorthy et al. (2020), ChemMedChem[1] | - | Au NPs | Au NPs decorated MOF | Photodynamic therapy, drug delivery, anticancer |
| Muhamed et al. (2023), Inorg. Chem.[2] | NU-1000 (Zr-based) | Au NPs | Au NPs grown on NU-1000 | Hydrogen evolution, photochemical and electrochemical catalysis |
| Huang et al. (2021), Chem. Comm.[3] | ZIF-8 | Ag nanowires | Core-shell Ag@ZIF-8 nanowires | $CO_2$ sensing |
| Liu et al. (2015), Adv. Mater.[4] | ZIF-8 | Ag nanowires | Core-shell Ag@ZIF-8 nanowires | Solar-driven butanol separation |
| Chen et al. (2025), Small Methods[5] | MOF-801 (Zr-based) | Plasmonic nanocrystals | MOF on plasmonic crystal composite | $CO_2$ photoreduction |
| Sun et al. (2025), Sens. Act. B | MIL-101 | Ag NPs | MIL-101@Ag with PDA layer | SERS enhancement |
| Li et al. (2018), Nano Res. | ZIF-8 | Au nanorods | Au NRs encapsulated in ZIF-8 | pH-triggered drug release and photothermal therapy |
| Li et al. (2019), J. Mater. Chem. A | HKUST-1 (Cu) | Ag NPs | Core–shell HKUST-1@Ag on SPCE | SERS detection of PAHs and 4-ATP |
| Zheng et al. (2016), Small | ZIF-8 | Au or Ag NPs | Core–shell (ZIF-8@NP) | SERS and molecular transport studies |

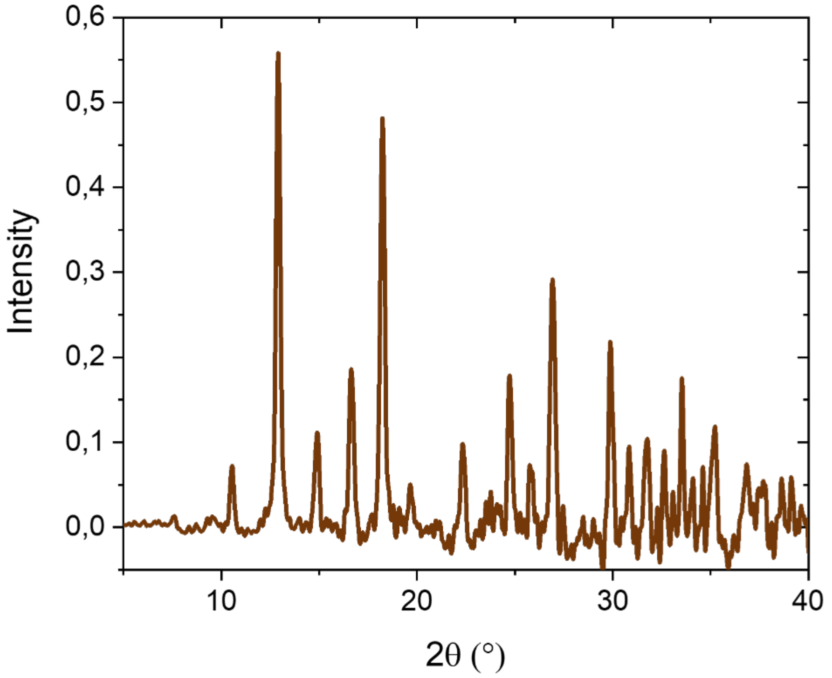


**Figure S1** XRD pattern of the ZIF-8 colloidal film.

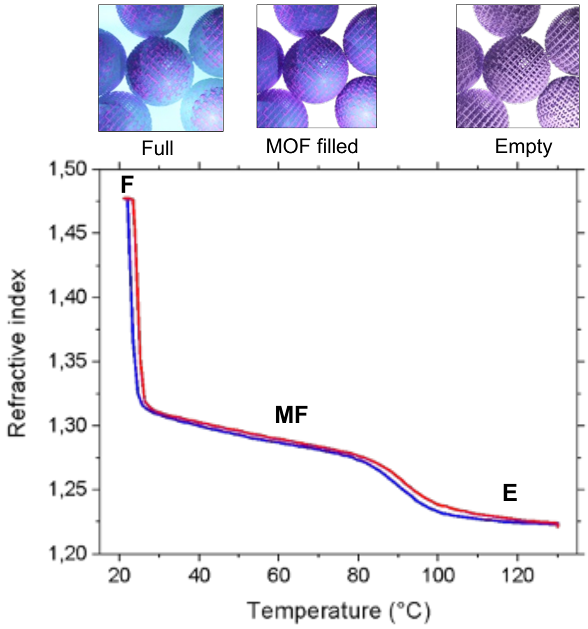


**Figure S2** Refractive index as function of the temperature of the ZIF-8 layer in presence of 0.7 $P/P_0$ of IPA.

**Calculation of the porous volume**

Starting from the values of refractive index associated to the three states E, MF and F we can evaluate the porous volume by using the Bruggemann effective medium approximation (BEMA) model (eq 1).[6] First, by taking the dielectric constant ε of the film in the empty (E) and filled state (F), this model allows the estimation of the dielectric constant of solid wall of the ZIF-8 material ($\varepsilon_{MOF}$). In the empty and full states the BEMA model can be written as follows:

$$f_{MOF}\frac{\tilde{\varepsilon}_{MOF}-\tilde{\varepsilon}}{\tilde{\varepsilon}_{MOF}+2\tilde{\varepsilon}}+f_A\frac{\tilde{\varepsilon}_A-\tilde{\varepsilon}}{\tilde{\varepsilon}_A+2\tilde{\varepsilon}}=0 \qquad \text{E state} \qquad (1)$$

$$f_{MOF}\frac{\tilde{\varepsilon}_{MOF}-\tilde{\varepsilon}}{\tilde{\varepsilon}_{MOF}+2\tilde{\varepsilon}}+f_W\frac{\tilde{\varepsilon}_{iso}-\tilde{\varepsilon}}{\tilde{\varepsilon}_{iso}+2\tilde{\varepsilon}}=0 \quad \text{F state} \qquad (2)$$

Where $f_{MOF}$ , $f_w$ and $f_A$ and $\varepsilon_{MOF}$, $\varepsilon_{iso}$, $\varepsilon_A$ the relative volumetric fractions and dielectric constants of three compounds MOF, Iso (Isopropanol) and A (Air). We consider the dielectric constants to be the square of refractive index values (real part of the dielectric constants). This assumption can be made for media that not absorb light in the considered range of wavelengths (typically 400-1000nm). Cauchy model for "transparent" materials was employed to fit ZIF-8 film.[7]

According to the scheme in Figure (2b), in the Filled state (F) all the air that was initially present in the film (porosity of the MOF + interparticle void) is replaced with isopropanol vapor. We can thus assume that:

$F_{Iso} = f_A$ (3)

that also corresponds to the total porous volume. According to Figure (2b), the refractive indices of the empty and filled states are 1.22 and 1.47 respectively. Considering the refractive indices of isopropanol and air equal to 1.37 and 1 respectively, the porous volume and the $\varepsilon_{MOF}$ (and the refractive index) can be calculated by combining equations (1), (2) and (3).

Following this calculation, the refractive index of the ZIF-8 wall is deduced to be equal to 1.63 while the total porous volume is 63%. By knowing these values, we can calculate the percentage of porosity associated to each family of mesopores by applying a 3 phase BEMA relation for intemediate states:

$$f_{MOF}\frac{\tilde{\varepsilon}_{MOF}-\tilde{\varepsilon}}{\tilde{\varepsilon}_{MOF}+2\tilde{\varepsilon}}+f_A\frac{\tilde{\varepsilon}_A-\tilde{\varepsilon}}{\tilde{\varepsilon}_A+2\tilde{\varepsilon}}+f_W\frac{\tilde{\varepsilon}_{Iso}-\tilde{\varepsilon}}{\tilde{\varepsilon}_{Iso}+2\tilde{\varepsilon}}=0 \qquad E < \text{state} < F \qquad (4)$$

In which:

$f_{MOF} + f_A + f_{Iso} = 1$ (5)

Since $f_{MOF}$ is equal to 0.37 this relationship can be rewritten as follows

$F_{Iso} = 0.63 - f_A$ (6)

and introduced in equation (4). From this calculation, the micropores and interparticle mesoporosities represent around 16% and 47% of the total porosity.

## FEM-Based Simulation Methods

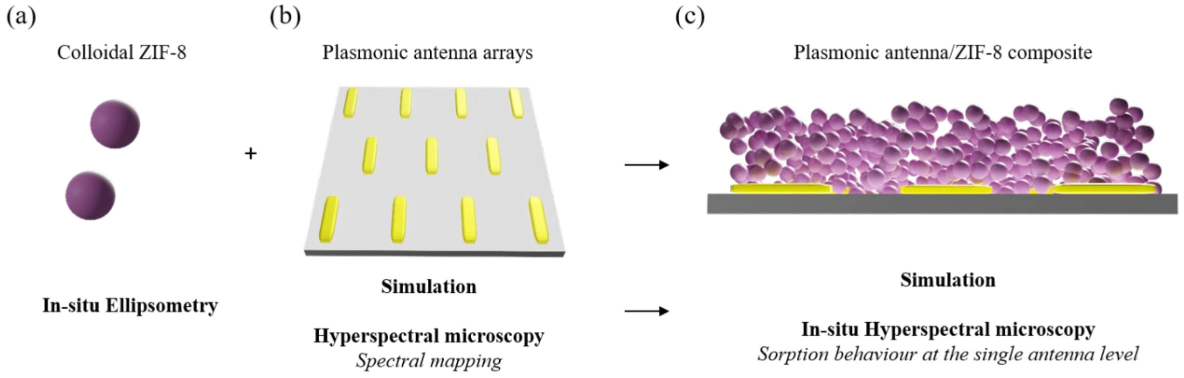


**Figure S3** Schematic of the experimental and simulated system using colloidal ZIF-8 nanoparticle and gold nanoantenna. The process involved several steps, including (a) synthesis and characterization of colloidal ZIF-8 nanoparticles with in-situ ellipsometry, (b) simulation and fabrication of gold nanoantenna using e-beam lithography, characterization with hyperspectral microscopy, and (c) deposition of colloidal film onto fabricated nanoantenna. The resulting system was studied with in-situ hyperspectral microscopy, including both experimental measurements and simulations.

We employed the Finite Element Method (FEM) to solve Maxwell's equations that describe the propagation of electromagnetic waves through complex structures. These equations are influenced by the geometry and material properties of the structure. The optical properties of the gold nanoantennas are described using the complex refractive index derived from the Johnson and Christy model.[8] As shown in figure S4 (a) the computational domains were created using CUBIT software, then meshed with triangles and simulated in MATLAB environment. For the ZIF-8 film, the medium is considered homogeneous, with a variable refractive index (n) obtained from the experimental ellipsometry data. We apply a plan wave with $E_x$ polarised and we studied the quarter of the entire structure. We added a perfectly matched layer (PML) in the X, Y and Z directions to enforce boundary condition and absorb outgoing waves without reflecting.

The FEM solution is obtained by transforming the PDEs into a system of algebraic equations. This process involves using finite elements to approximate the solution over the domain. The obtained equations are the Helmholtz equation for the diffracted light. For a three-dimensional simulation with transverse electric (TE) polarization, the FEM formulation with edge elements is given by the following wave equation:

$$\nabla \times \left(\frac{1}{\mu_r} \nabla \times \boldsymbol{E_d}\right) = k_0^2 \epsilon_r \boldsymbol{E}_d + k_{\boldsymbol{0}}^2 (\epsilon_r - \epsilon_b) \boldsymbol{E_{Inc}}$$

Where, $\boldsymbol{E_d}$ is the electric field in the domain, $k_0$ is the free space wavenumber, $\epsilon_r$ is the relative electric permittivity, $\epsilon_b$is the background permittivity and $\mu_r$ is the relative magnetic permeability

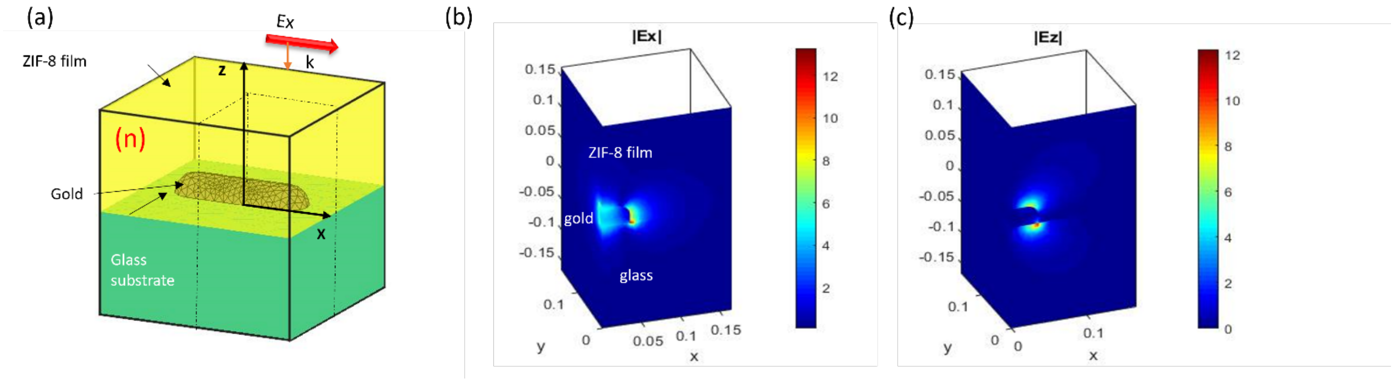


**Figure S4** (a) the simulated system with a gold nanoantenna placed on top of a glass substrate, with the nanoantenna surrounded by a homogeneous layer. This layer's refractive index is varied in the simulation to model different experimental conditions for the adsorption/desorption process in zif-8 film with isopropanol vapor. (b,c) the distribution of the electric field of the gold nanoantenna along the x and z components, respectively.

By following this weak formulation and discretizing the problem using edge elements, we can obtain the electric field distribution in the structure (Figure (S4 b-c) along the x,y and z component for single antenna. The scattering cross-section is determined based on the calculations of the extinction and absorption cross-sections as follows:

$$\sigma_{Scat} = \sigma_{Ext} - \sigma_{Abs}$$

The absorptivity (A) is calculated as the ratio of the absorbed power $P_{Ads}$ to the incident power $P_{Inc}$. The absorbed power is obtained by summing the absorption cross-sections $\sigma_{Abs}$ of individual antennas and multiplying by the incident power density $\sigma_{Abs}$. The formula for absorptivity is:

$$A = \frac{P_{Ads}}{P_{Inc}} = \frac{(\Sigma\, \sigma_{Abs} \, * \, I_{Inc})}{P_{Inc}}$$

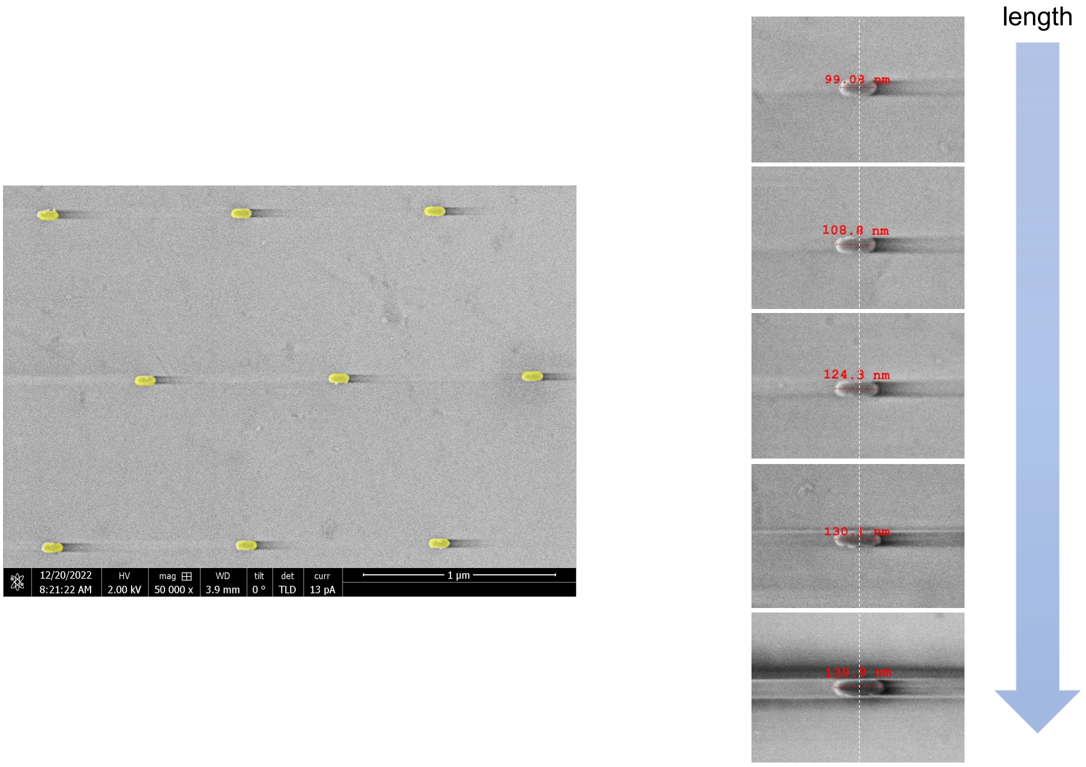


**Figure S5** SEM image of an array of nanoantenna; in the left with a length of around120nm and periodicity of 1µm that we used for our study. In the right: SEM images of various nanoantenna fabricated with lengths ranging from 90nm to 130nm.

The nanoantennas were fabricated using electron beam lithography (EBL). Arrays of antennas with lengths ranging from 90nm to 130nm with a width of 30 nm were created to determine the optimal geometry for achieving a specific resonance. By placing the ZIF-8 film on top of these antenna, we aim to identify the array that resonate at a wavelength of 850nm when the film is in its fully adsorbed state.

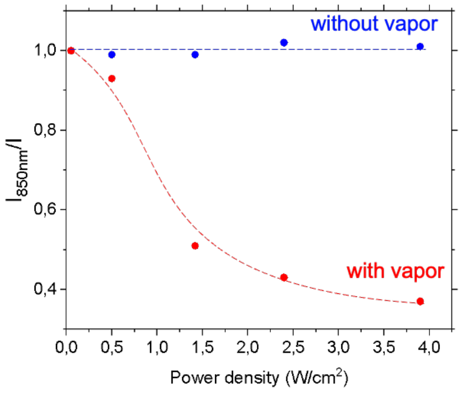


**Figure S6** Evolution of the normalized scattering intensity at 850 nm as function of the incoming light intensity (the dotted lines are a guide for the eye).

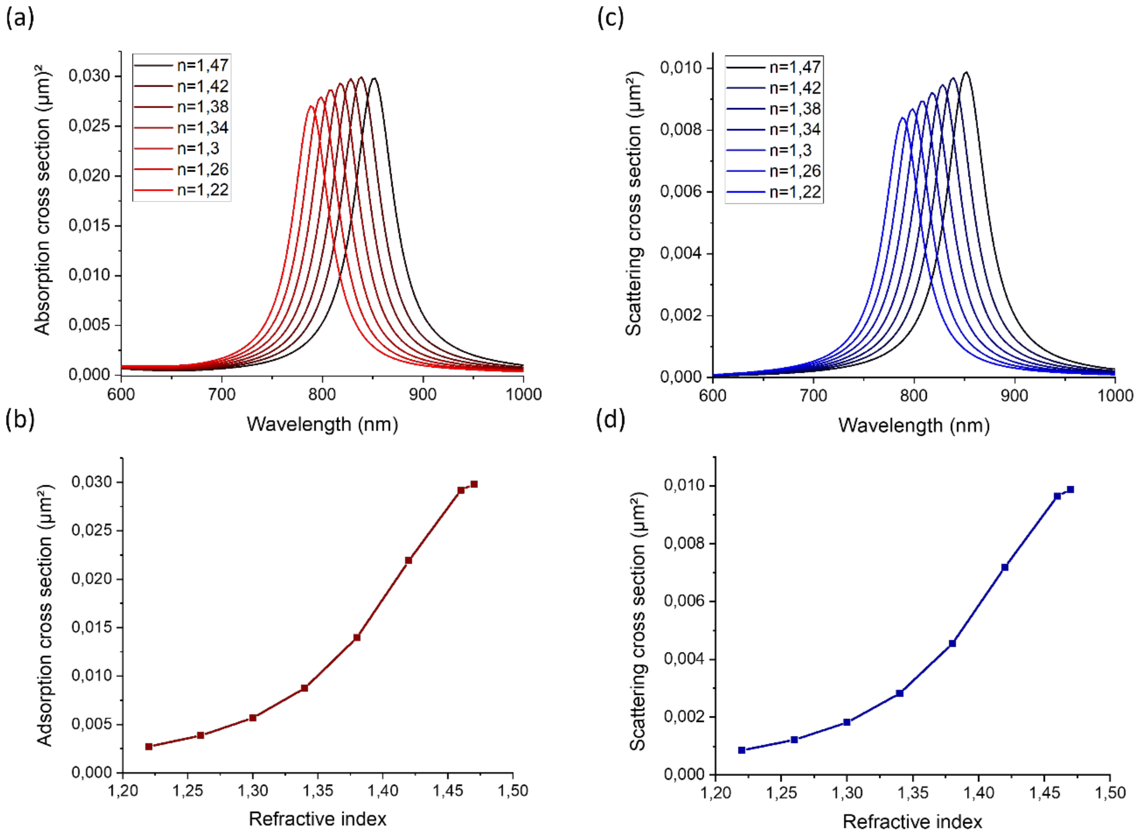


**Figure S7** Simulation results indicating the correlation between the evolution of absorption and scattering at 850nm for varying refractive index of the MOF layer: (a) the adsorption cross-section of the gold nanoantenna as function of wavelength for different refractive index of the surrounding medium. (b) the adsorption cross-section as a function of the refractive index for a single wavelength of 850 nm. (c) the scattering cross-section of the gold nanoantenna as function of wavelength for different refractive index of the surrounding medium. (d) the scattering cross-section as a function of the refractive index for a single wavelength of 850 nm.

**Estimation of the local temperature**

To estimate the local temperature , we compared the reduction in normalized scattering at 850 nm, shown in Figure 4, with the normalized scattering at 850 nm as a function of temperature used as a calibration curve.[9] This curve was derived by simulation, starting from the experimental values of the refractive index of the ZIF-8 layer as a function of temperature shown in Figure 2(b).

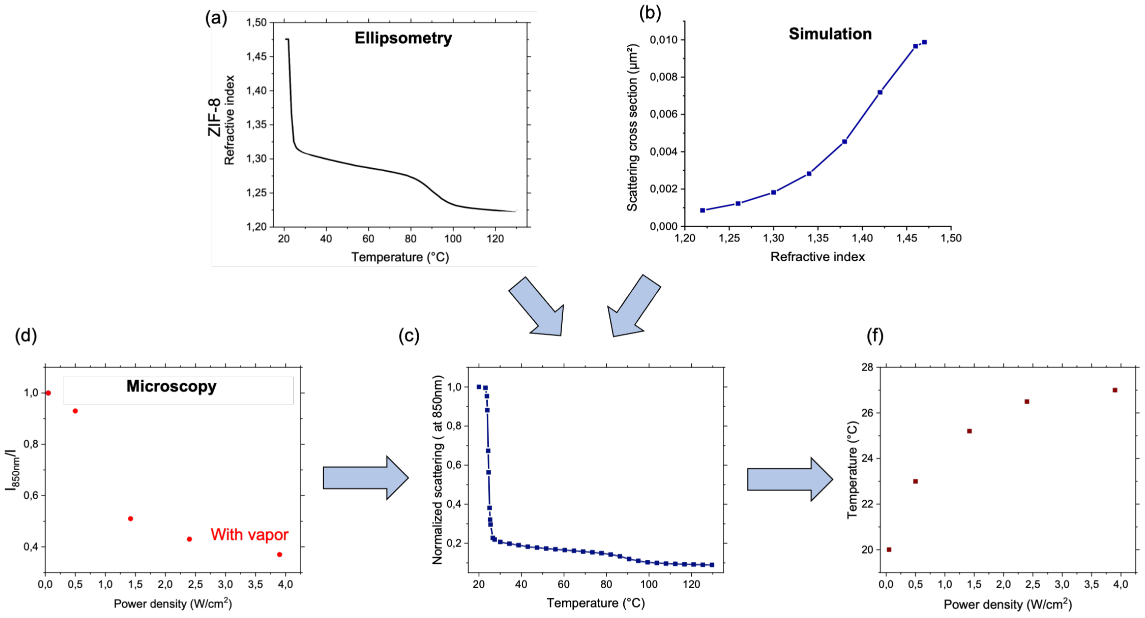


**Figure S8** (a) Evolution of the refractive index as a function of the temperature in the presence of 0.7 $P/P_0$ of IPA for the desorption isobar measured by in situ ellipsometry. (b) scattering cross-section as a function of the refractive index for a single wavelength of 850 nm (c) Simulated evolution of the normalized scattering at 850nm as a function of the temperature (d) Evolution of the normalized scattering intensity at 850 nm as a function of the incoming laser power obtained experimentally ; (f) temperature evolution as a function of the laser power density

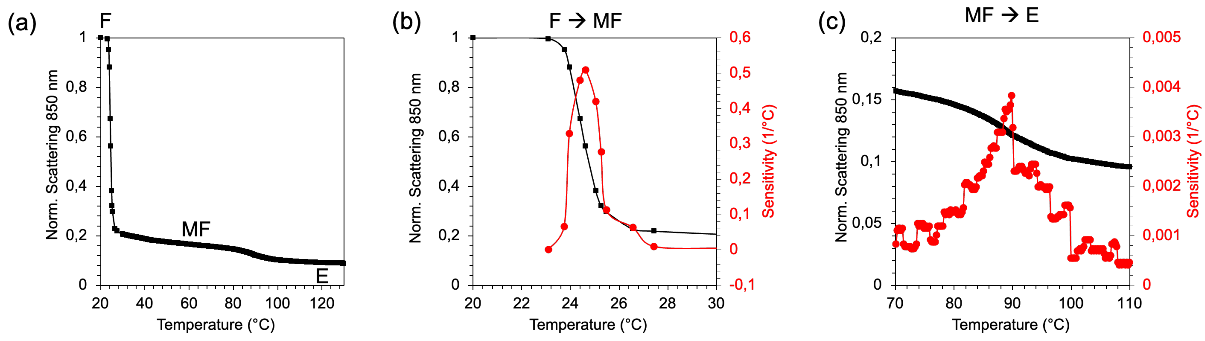


**Figure S9** (a) Evolution of the scattering intensity at 850 nm as a function of the temperature in the presence of 0.7 $P/P_0$ of IPA for the desorption isobar; (b) and (c) evolution of the sensitivity as function of the temperature for two temperature ranges.

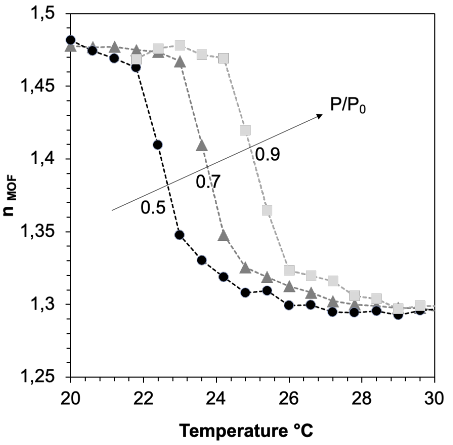


**Figure S10** Evolution of the refractive index of the ZIF-8 layer as function on the temperature for three vapor pressures.

**Dynamic analysis of independent antennas: experimental details and autonomous image analysis**

The array of MOF-coated antennas was illuminated by an 850 nm laser through a condenser to ensure uniform illumination. The analysis was conducted with constant laser power over varying time intervals, progressively increasing and then decreasing the power. The results presented in the main text were obtained using a specific interval of laser powers (determined experimentally) at which the antennas exhibited the most prominent oscillations. Reducing the laser power too much prevented the evaporation of IPA around the antennas, inhibiting their oscillations. Conversely, using excessive laser power caused excessive heating of the antennas, which shifted the plasmonic peak and kept the area around them dry.

To analyze the oscillations, we captured images using the same microscope as for hyperspectral imaging, but with a standard camera instead of a hyperspectral detector. This camera is slightly sensitive to the 850 nm light scattered by each particle. Since the presence of MOF around the particles increases light scattering, as seen in Figure 5 of the main text, we were able to observe the filling and emptying cycles of each antenna, which were sufficiently spaced to be evaluated individually using optical methods. The array was not entirely uniform due to the detachment of some antennas during previous failed MOF deposition attempts. In fact, this irregularity was advantageous for image analysis, as it facilitated the identification of specific antennas during processing, minimizing errors.

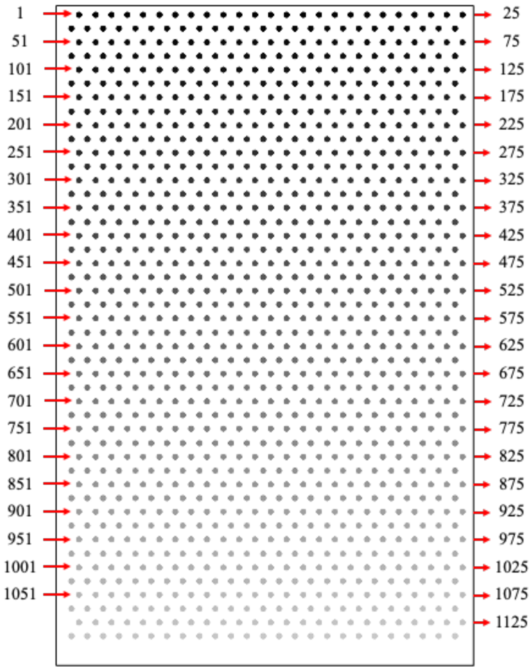


**Figure S11** Supporting image to allow identify the antennas by gray-value. The numbering of each antenna is obtaining “reading” the set of antennas as normally done with a book. From left to right from top to bottom.

Figure S8 provides a “map” linking each particle to its corresponding number, enabling the reader to match the particle numbers shown in the titles of our plots with their actual locations in the image. The gray value of each circle corresponds to the antenna number. This facilitates a comparison of oscillations between nearby particles and may help identify patterns that might have been overlooked in our analysis. Based on our observations, the particles appear to behave as though they are isolated, not only optically but also "environmentally," meaning that the drying of MOF around one particle does not influence the behavior of neighboring particles. Consequently, we did not observe any collective behaviors or particles synchronizing in phase, as might be expected if the spacing between them were reduced. This will be explored in future publications.

To begin our analysis, we stabilized the "raw" data, which consisted of a series of images captured every second, using a custom tracking and stabilization algorithm. The algorithm translates the images by the number of pixels determined by the tracking process. In cases where the antennas' XY movement was too rapid, manual tracking was applied for a few frames.

No Z-axis stabilization was performed since we only had images of a single focal plane. However, the focus was maintained consistently throughout the experiment.

The luminosity of the scattered light from each particle was analyzed using a straightforward algorithm based on the "Plot Z-Axis Profile" function in ImageJ, as described below. This macro allowed us to capture both the average luminosity and the individual luminosity of each antenna in a single plot. By using the "data" button and selecting the "copy all data" option, we exported the scattering values as numerical data into an Excel file.

The data, in .xlsx format, were then imported into a Mathematica notebook, where they were organized based on laser power, creating lists of lists, each containing data for all particles with $n$ time points (depending on the duration the specific laser powers were applied, with transient periods between power changes excluded). The scattered intensity values were then fitted to a straight line, and the value of this

linear fit was subtracted from the intensity data. This step was performed to eliminate any drift in intensity, thereby making the values comparable by centering them around zero.

To continue the analysis, the oscillation data were normalized by dividing by the absolute value of the maximum or minimum of the linearly adjusted intensities, restricting the oscillations between 1 and -1. The normalized time series were then processed using a numerical function in Mathematica to compute their Fourier transform. Since the time series had different numbers of data points, the frequency plots had varying spacings between frequency values, calculated as ½*2π/T, where $T$ is the total time of data acquisition at a given laser power.

**XY Tracking Algorithm explanation:** The tracking algorithm processes a video that includes a few antennas to minimize computation time. The user selects one antenna, which is assumed to remain within the video frame throughout. Since the antenna's position corresponds to the maximum brightness of its scattered light, the algorithm isolates it by multiplying each frame with a secondary frame that has a Gaussian distribution of brightness centered on the maximum from the previous frame. This helps to avoid tracking errors caused by unwanted optical effects or sudden movements. For each multiplied frame, the position of the maximum is identified and recorded. The displacement of the maximum in each frame, relative to the first frame, is then calculated and used in the subsequent stabilization process. The algorithm was implemented as an ImageJ macro, and its code is provided here. The program generates the list of the displacements in the "Log" of imageJ. Expected values of "displacements" are between -2 and +2. This list has to be saved in a text file (.txt) that has to be given as input to the "moving" algorithm.

**XY Tracking Algorithm ImageJ macro Code:**

```
ImgID=getImageID();
rename("Source");
Name=getTitle();
getDimensions(width, height, channels, slices, frames)

MaxIntX=101; // X-position antenna we want to track
MaxIntY=127; // Y-position antenna we want to track
Sigma=6;    // "radius" of the gaussian
FindSpot(MaxIntX,MaxIntY,slices,Sigma,Name);

function FindSpot(MaxIntX,MaxIntY,slices,Sigma,Name) {
	MaxX=MaxIntX;
	MaxY=MaxIntY;
	for (n = 1; n < slices+1; n++) {
		//print("ok", n);
		selectWindow(Name);
		setSlice(n);
		run("Make Substack...", "  slices="+n);
		DrawGaussian(MaxX,MaxY,Sigma,width,height);
		imageCalculator("Multiply", "Substack ("+n+")","Gauss");
		selectWindow("Substack ("+n+")");
		run("Find Maxima...", "noise="+(width+height)+" output=List");
		MaxX = getResult("X",0 );
		MaxY = getResult("Y",0 );
		if (n==1) {
			MaxXstart=MaxX;
			MaxYstart=MaxY;
		}
```

```
			print(MaxX-MaxXstart,",", MaxY-MaxYstart);
			selectWindow("Substack ("+n+")");
			close();
			selectWindow("Gauss");
			close();
		}
}

function DrawGaussian(CenterX,CenterY,Sigma,width,height) {
		newImage("Gauss", "32-bit black", width, height, 1);
		for (i = 0; i < width; i++) {
			for (j = 0; j < height; j++) {
				//ValInput= getPixel(i, j);
				ValInput=1;
				PosX= i-CenterX;
				PosY= j-CenterY;
				Value =ValInput*exp(-1*(PosX*PosX+PosY*PosY)/2/Sigma/Sigma);
				setPixel(i		, j, Value);
			}
		}
}
```

**XY Translate Algorithm explanation:** The algorithm simply takes the values obtained by the previous algoritm saved in the .txt file (please put the right folder in the macro) and uses them in the “Translate” function of ImageJ. It proceeds in doing so slice by slice (timepoint by timepoint).

**XY Translate Algorithm ImageJ macro Code:**

```
ImgID=getImageID();
rename("Source");
Name=getTitle();
FilePathName="	"; //put filepath of your .txt file with obtained by the previous algoritm

print("\\Clear");
TranslateXY(FilePathName);

function TranslateXY(FilePathName) {
		getDimensions(width, height, channels, slices, frames);
		RetArrayXY = parse_moduli(FilePathName);
		deltind=RetArrayXY.length/2;
		if (frames==1) {
			frames=slices;
			slices=1;
		}
		for (i = 1; i < slices*frames+1; i++) {
			setSlice(i);
			temp=floor((i-1)/slices);
			run("Translate...", "x=" + (-RetArrayXY[temp]) +" y="+ (-RetArrayXY[temp+deltind])
+ " interpolation=None slice");
			print(i,temp,-RetArrayXY[temp],-RetArrayXY[temp+deltind]);
		}
}

function parse_moduli(FilePathName) {
//parse the moduli file
```

```
	moduli_file = File.openAsString(FilePathName);
	moduli_lines = split(moduli_file, "\n");
	moduliX = newArray(moduli_lines.length);
	moduliY = newArray(moduli_lines.length);
	for (m=0; m<lengthOf(moduli_lines); m++){
		moduli_n= split(moduli_lines[m], ";,");
		moduliX[m] =   parseFloat(moduli_n[0]);
		moduliY[m] =   parseFloat(moduli_n[1]);
	}

	RetArray=Array.concat(moduliX,moduliY);
	return RetArray;
}
```

**Scattering intensity extraction algorithm:** The intensity extraction algorithm processes the video, which contains hundreds of antennas arranged in hexagonal symmetry. The "Plot Z-Axis Profile" function is applied to each antenna, following the numbering provided in Figure SX. It is crucial to accurately define the distance between the antennas to ensure that the analysis is correctly centered on each one. Additionally, the reader has to specify the radius when drawing the circle for the "Plot Z-Axis Profile" that should be approximately half the radius of the antenna's scattering zone to minimize the impact of XY vibrations, which can still affect the video at the pixel level.

After performing the "Plot Z-Axis Profile," the program draws a circle over the analyzed area, allowing the user to verify that the particle has been properly centered. All intensity profiles are compiled into a single plot, and the data can be exported into Excel for further analysis. By setting to 1 the value "drawing" it is possible to ask the program to fill the circles with a gray value corresponding to the numbering of the antenna (which will be useful for the following analysis with Mathematica). This algorithm was implemented as an ImageJ macro, and the code is provided here.

**Scattering intensity extraction ImageJ macro code:**

```
ImgID=getImageID();
rename("Source");
Name=getTitle();

FirstX= -1; // X position of the upper left corner of the oval corresponding to the first particle
FirstY= -10; // Y position of the upper left corner of the oval corresponding to the first particle
OvalRad=10; // radius of the oval used to analyze the particles.
distance_particles=23.24;

zprofiling=1; // deactivate (=0) this to oly perform the numbering of the antennas
drawing=1; // activate (=1) this if you want to draw a circle with color values equal to the antenna number after having done the Z profile. Useful for double checking the antenna number

RefX=FirstX;
RefY=FirstY;
if (zprofiling == 1) {
		run("Plot Z-axis Profile");			// the first profile takes the average of the full image and produces a plot with the name Source-0-0 that is useful to be selected
}
setBatchMode(true);

N_particles_X=25;  // N of particles in the X direction
N_particles_Y=30;  // N of particles in the Y direction
```

```
counter=0;

for (j = 1; j < N_particles_Y+1; j++) {
	CorrX=0.5*distance_particles;
	if ((j/2) == floor (j/2)) {
		CorrX=0;
	}
	for (i = 1; i < N_particles_X+1; i++) {
		CenterOvalX=FirstX+i*distance_particles;
		CenterOvalY=FirstY+j*distance_particles*sqrt(3/4);
		if (zprofiling == 1) {
			print(CenterOvalX+CorrX,CenterOvalY);
			MeasZprof (CenterOvalX+CorrX,CenterOvalY,OvalRad,Name,ImgID);
		} else {
			makeOval(CenterOvalX-OvalRad/2+CorrX,		CenterOvalY-OvalRad/2,
OvalRad, OvalRad);
			if (drawing == 1) {
				Color.set(j*N_particles_X+i)
				Color.set(counter)
				run("Fill", "slice");;
			}
		}
		run("Draw", "slice");
		counter=counter+1;
	}
}
setBatchMode(false);

function MeasZprof (PosXset,PosYset,Diam,Name,ImgID) {
	PosX=PosXset-Diam/2;
	PosY=PosYset-Diam/2;
	selectImage(ImgID);
	makeOval(PosX, PosY, Diam, Diam);
	run("Plot Z-axis Profile");
	selectWindow(Name+"-"+0+"-"+0);
	//print(PosX,PosY);
	Plot.addFromPlot(Name+"-"+floor(PosX)+"-"+floor(PosY), 0);
	selectWindow(Name+"-"+floor(PosX)+"-"+floor(PosY));
	close();
	selectImage(ImgID);
	makeOval(PosX, PosY, Diam, Diam);
	run("Draw", "slice");
}
```

**Fourier transform and data selection:** The scattered intensity values from each antenna are stored in an Excel file, which is opened in Mathematica for analysis. The first row and the first two columns of the file are discarded, as they contain the antenna names, slice numbers, and the average intensity of the full image, respectively. The remaining data are split into different series, each corresponding to a different laser power, allowing for easier data manipulation. Data collected while the laser power was being changed are excluded from the analysis.

Each data series, which contains a different number of data points (collected at one-second intervals), is normalized to the corresponding laser power. Then, any linear trend or average value in the data—due to small variations in focusing or camera efficiency over time—is removed. This step does not affect the Fourier transform of the antenna oscillations. After removing the linear trend, the data are normalized to the maximum or minimum value within each series, resulting in data values that range between -1 and 1.

The Fourier transform algorithm is then applied to the normalized data. The output provides the intensity of the oscillations at different frequency intervals for each series. It is important to note that the resolution of the X-axis (frequency) in the Fourier transform is determined by the inverse of the sampling rate (in this case, 1 image per second) multiplied by the inverse of the total number of data points collected. This means that series with longer observation periods offer better frequency resolution. For more detailed understanding of the discrete Fourier transform algorithm, readers should refer to Mathematica and other related documentation.

In "ALL-Antennas.doc", we present the time-dependent normalized scattered intensity (black) alongside its Fourier transform (red). The axes are color-coded to correspond with each value. For some particles, distinct peaks are visible in the Fourier transform, while for others, this is not the case. This analysis enables straightforward identification of the oscillating antennas sorting them as respect to the frequency, light power...As an example, in Figure S10, 11 and 12 we show the top 9 particles oscillating at different frequency under laser power 2.4W cm$^{-2}$.

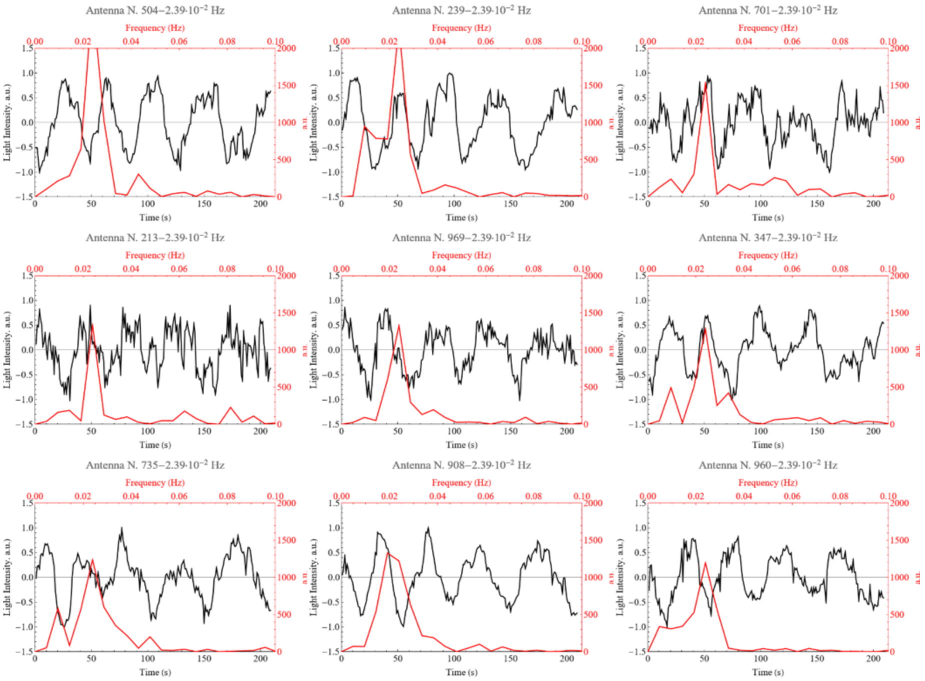


**Figure S12** Best particles oscillating at 2.39 10$^{-2}$ Hz under laser power 2.4W cm$^{-2}$

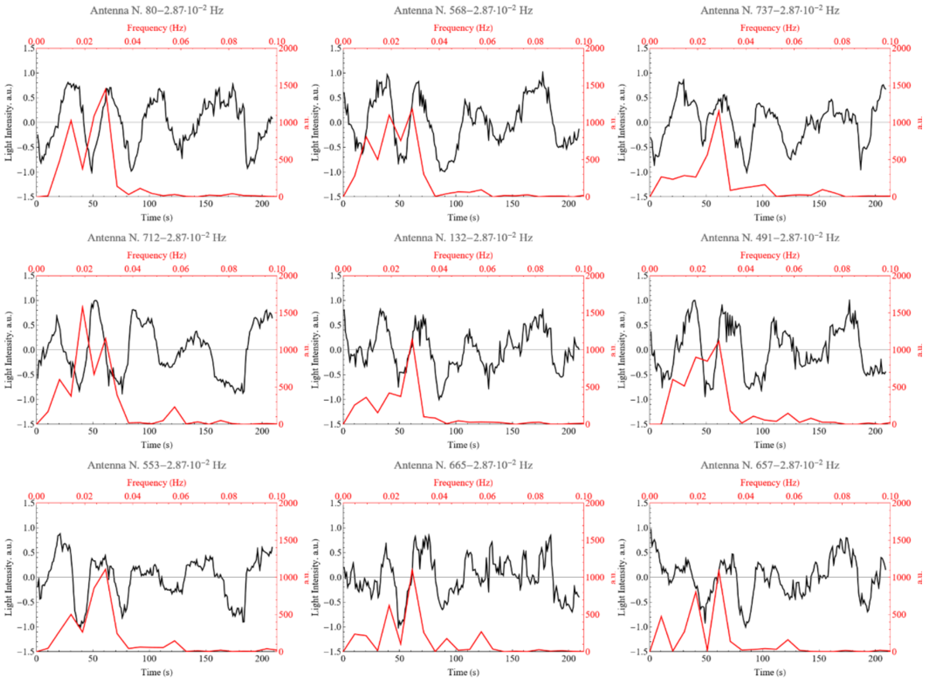


**Figure S13** Best particles oscillating at 2.87 $10^{-2}$ Hz under laser power 2.4W $cm^{-2}$

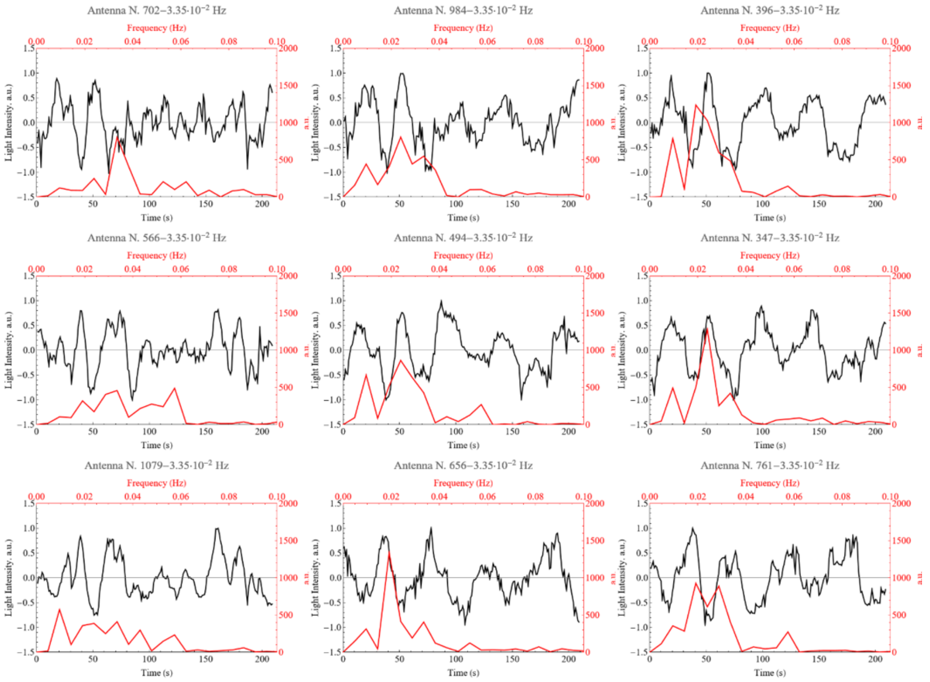


**Figure S14** Best particles oscillating at 3.35 $10^{-2}$ Hz under laser power 2.4W $cm^{-2}$

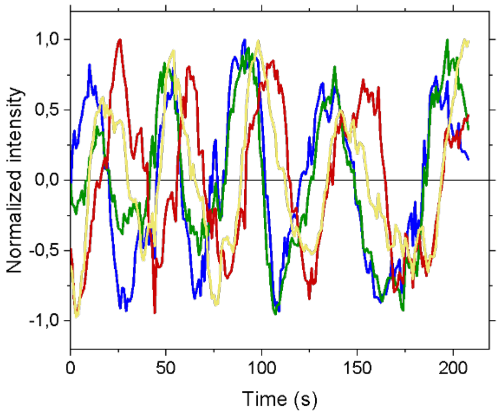


**Figure S15** Scattering evolution of four antennas not located close to each other exhibiting a similar frequency but not in phase

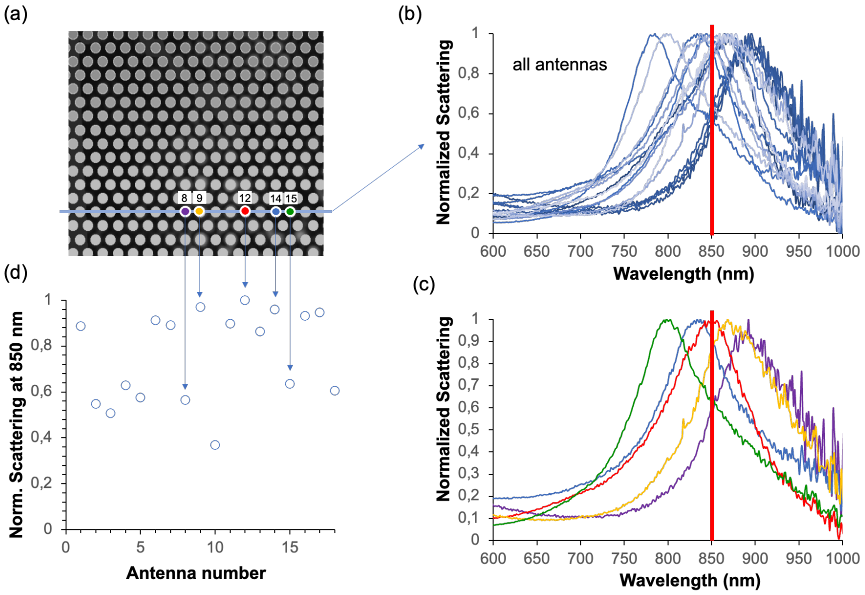


**Figure S16** (a) Scheme of the array, (b) scattering intensity curve of a row of antenna/ZIF-8 in presence of vapor. (c) selected scattering spectra of antenna/ZIF-8s. (d) Evolution of the scattering intensity at 850 nm for a row of antenna/ZIF-8s.

## Thermal simulation

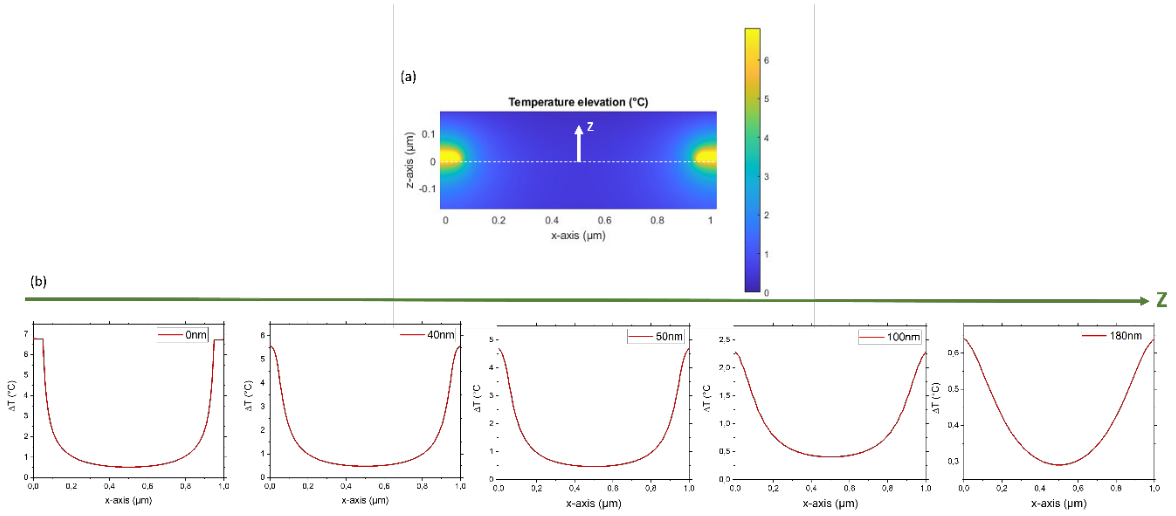


**Figure S17** (a) Heat map of temperature elevation for two nanoantenna with a periodicity of 1µm, illustrating the temperature distribution along the x-axis (0 to 1µm) and the z-axis (0 to 0.2 µm). The temperature distribution in a 2D plot, where the color intensity indicates the level of temperature elevation. (b) Series of line plots indicating the temperature distribution along the x-axis for different z-values (z= 0, z=40nm, z= 50nm, z=100nm and z=180nm respectively). Each plot shows the temperature profile through the two nanoantennas

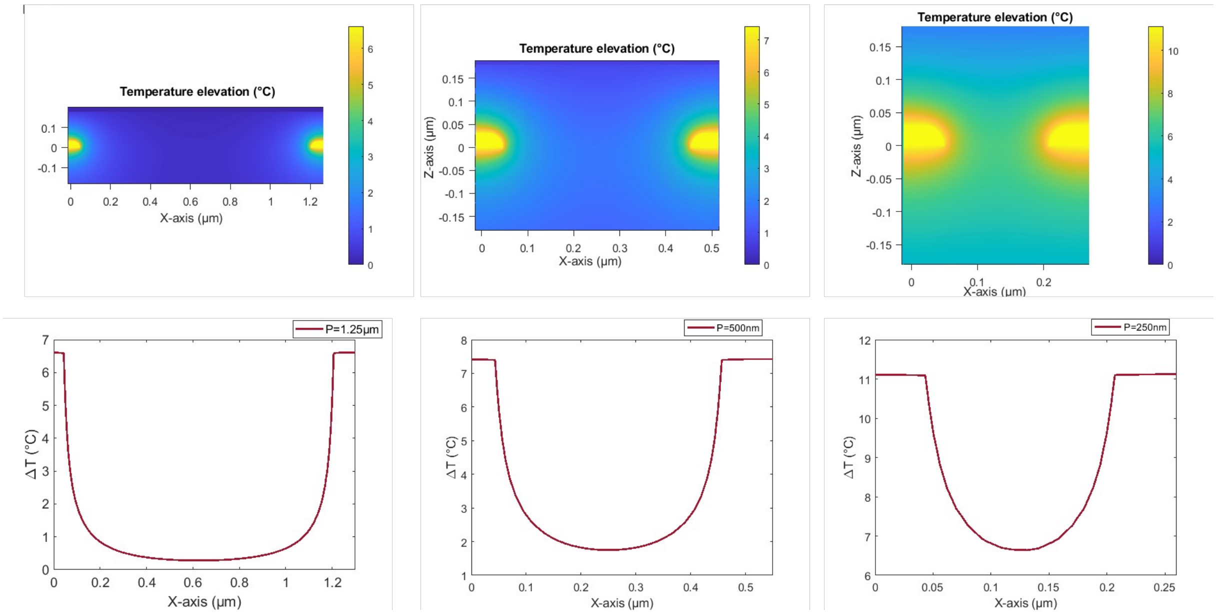


**Figure S18** Heat map and line plots of temperature elevation for two nanoantenna placed at different distances

For the thermal simulation, we accounted for the power of heat generation $Q_v$ (power density) inside the nanostructure. This power is directly proportional to the Absorptivity A calculated theoretically , $P_L$ laser power or the incident light and the nanoantenna volume V, with:

$$Q_v = \frac{A * P_L}{V}$$

We specified the thermal conductivity values for the materials in each domain. For the gold antenna, the thermal conductivity was set to 318 W/m·K. The simulation was carried out in a 3D domain with the following properties:

| **Material** | **Thermal Conductivity (W/m·K)** | **Specific Heat Capacity (J/m³·K)** | **Mass Density (kg/m³)** |
|---|---|---|---|
| Gold (Au) | 318 | $2.472 \times 10^6$ | $19.32 \times 10^3$ |
| Air | 0.0259 | $1.22 \times 10^3$ | $2.2 \times 10^3$ |
| Substrat | 1.6478 | $1.6478 \times 10^6$ | $2.2 \times 10^3$ |
| ZIF-8 Film | 0.3 | $8.84 \times 10^5$ | $0.94 \times 10^{-3}$ |

The thermal conductivity values for ZIF-8 were examined in the study of Wei et al,[10] it was revealed that thermal conductivity of ZIF-8 increases linearly from 0.165 W/m.K to 0.319 W/m.K during ethanol adsorption, where ethanol has a thermal conductivity of 0.17 W/m.K. Given that the thermal conductivity of isopropanol is 0.15 W/m.K, we can estimate the increase in thermal conductivity of ZIF-8 when isopropanol is adsorbed by applying the linear relationship, we estimate that the thermal conductivity could increases to approximately 0.3 W/m.K. We only considered a convective heat transfer at the top and the bottom of studied domain where the convective coefficient is equal to 20 (W/m²·K). The volumetric heat sources of gold nanoantenna were applied using a Gaussian distribution to represent the heat generation more accurately and equal to $1.5 \times 10^{-2}$. The initial temperature was set to 293.15 K. Boundary conditions were established using Dirichlet conditions to simulate realistic constraints with (Dirichlet ($T = T_0$) = 293.15)

We then computed the global matrices for the thermal system and incorporated the heat sources. The stationary thermal system evolution was solved to obtain the temperature distribution within the nanoantenna array.

**Kinetic analysis**

We have performed experiments of both plasmonic heating rate and adsorption/desorption rate in the MOF layer to estimate the characteristic timescales of the involved processes.

In the first experiment, we focused on the plasmonic heating rate. We simulated the transient thermal behavior of plasmonic gold nanoantennas subjected to localized photothermal excitation. The heat dissipation dynamics were modeled using a lumped capacitance approach, assuming that the temperature within the gold volume remains spatially uniform during heating due to its high thermal conductivity. The thermal time constant τ of the gold structure was calculated as:

$$\tau = \frac{\rho\, C_p\, V}{h\, A}$$

where ρ is the mass density of gold, $C_p$ its specific heat capacity, V its volume, A the effective heat exchange surface area, and $h$ the convective heat transfer coefficient. The absorbed power was inferred from the maximum temperature rise experimentally observed, and the temperature evolution over time was fitted using an exponential function of the form:

$$T(t) = T_{max}(1 - e^{-t/\tau})$$

Where $T_{max}$ is the experimentally measured maximum temperature increase. In this case, the plasmonic heating is very fast with a time constant of 63 µs.

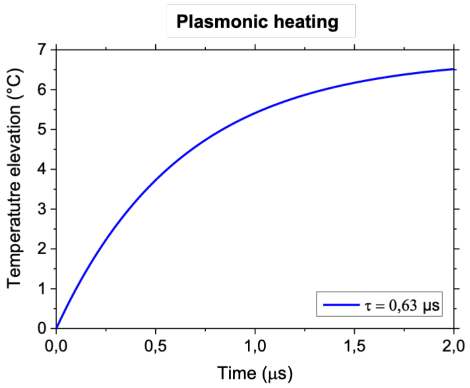


**Figure S19** Evolution of the temperature of the plasmonic antenna upon light irradiation

The second experiment aimed at evaluating the characteristic sorption time constant of the MOF. For this purpose, we fabricated a simplified sample consisting of a glass substrate coated with spherical gold nanoparticles, chosen for their scalability and ease of use (as reported in [11] and [12]). In this context, we only required a comparable heat source, and spherical nanoparticles provided a practical alternative, since the fabrication of plasmonic nanoantennas is less easily transferable and would require advanced techniques such as electron beam lithography.

The plasmonic nanoparticles were coated with the MOF layer, and we monitored the dynamic evolution of the MOF's refractive index over time upon laser excitation (532 nm). As the laser is applied, the refractive index of the MOF decreases, indicating that desorption occurs due to local heating. For clarity, we present the absolute value of the change in refractive index versus time.

From this response, we were able to extract a characteristic time constant of approximately 20 seconds, which give an indication the sorption kinetics under plasmon-induced thermal stimulus.

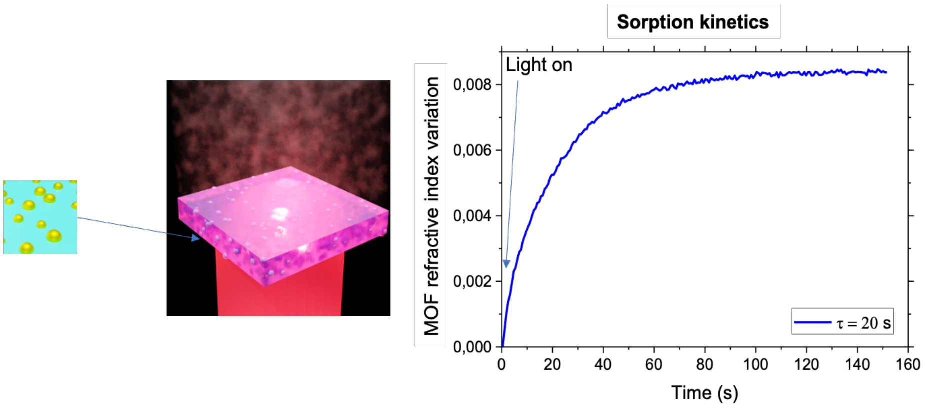

**Figure S20** Sorption kinetics : (absolute value) variation of the refractive index of the MOF layer upon light irradiation measured by ellipsometry

**Dynamic simulation**

Based on experimental kinetic parameters and hysteresis behavior of the MOF layer, we developed a simple hysteresis-based model that reproduces the self-oscillatory behavior. This model allowsrunning simulations to systematically explore the influence of various parameters, in particular laser intensity and hysteresis shape, on the oscillation dynamics.

We considered our system as a material whose optical absorptivity depends on temperature, with a hysteretic response and a finite time constant (soprtion time constant) governing its evolution. In particular, the absorptivity exhibits different transition thresholds during heating and cooling due to the hysteresis of the F to MF states reported in Figures 2.

*Physical Model :* The system under consideration consists of a material with an initial absorptivity, which absorbs optical power from a constant incident optical flux. The temperature of the material changes as a result of both the absorption of incident optical power and thermal dissipation to the environment. The material is assumed to be in thermal equilibrium with the surroundings, and the thermal behavior of the system is modeled using differential equations derived from energy conservation principles.

*Governing Equations :* The thermal behavior of the system is modeled by two coupled differential equations: one for the temperature, T(t) and one for the absorptivity, α(t). These equations describe the evolution of temperature and absorptivity over time based on the incident power, the thermal dissipation, and the feedback between temperature and absorptivity. The rate of change of temperature, dT/dt is given by the following equation derived from energy conservation:

$$\frac{dT}{dt} = \frac{\alpha(t)P_{\text{in}} - k(T - T_{\text{env}})}{C}$$

where:

- $P_{in}$ is the incident optical power,
- α(t) is the time-dependent absorptivity of the material.
- k is the thermal dissipation coefficient,
- $T_{env}$ is the ambient temperature
- C is the heat capacity of the material.

The absorptivity, α(t), evolves over time as a function of temperature. A sigmoid function is used to model the temperature-dependent change in absorptivity with different transition thresholds and steepness for heating and cooling phases. The absorptivity evolves with a delayed relaxation toward this equilibrium value with a characteristic time constant τ (20s as determined experimentally).

$$\frac{d\alpha}{dt} = \frac{\alpha_{eq}(T, \text{mode}) - \alpha}{\tau_\alpha}$$

where mode switches between heating and cooling depending on the sign of the temperature derivative, enforcing the hysteresis. The absorptivity follows different paths during heating and cooling phases, with transitions occurring at specific threshold temperatures.

- Heating Phase: When the system is in the heating mode, the absorptivity increases as the temperature rises.
- Cooling Phase: When the system switches to cooling mode (due to a decrease in temperature), the absorptivity decreases as the temperature drops.

The temperature dependence of absorptivity was modeled using sigmoidal functions to capture the smooth yet sharp transition between low and high absorptivity states near threshold temperatures. These functions provide a convenient phenomenological description of hysteresis by defining separate transition branches for heating and cooling.

For each mode (heating or cooling), the equilibrium absorptivity is given by:

$$\alpha_{eq}(T) = \alpha_{min} + \frac{\alpha_{max} - \alpha_{min}}{1 + \exp\left[\beta(T - T_{th})\right]}$$

where:

- $\alpha$ min and max are the lower and upper bounds of absorptivity.
- $T_{th}$ is the threshold temperature at which the transition occurs.
- $\beta$ is a steepness parameter controlling how sharply the transition happens.

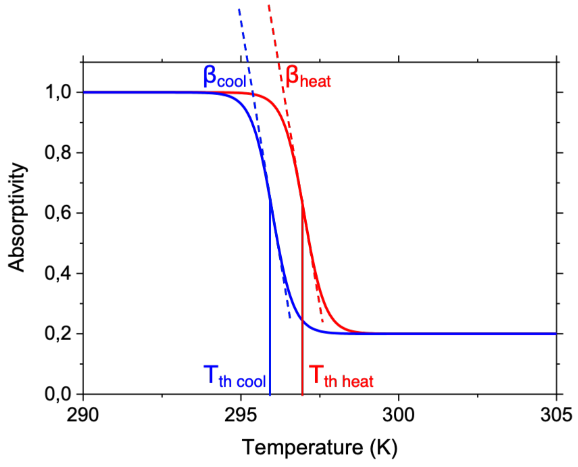


**Figure S21** Absorptivity vs temperature hysteresis curve modeled using sigmoidal functions

For the simulation we used values transition thresholds, $\beta$ , $\alpha$ min and max and time constant determined experimentally. For the other parameter values (heat capacity, dissipation coefficient, incident power) we imposed physically reasonable values to explain the observed experimental behavior. The ultimate goal is to propose a mechanism and gain indications about the influence of various parameters.

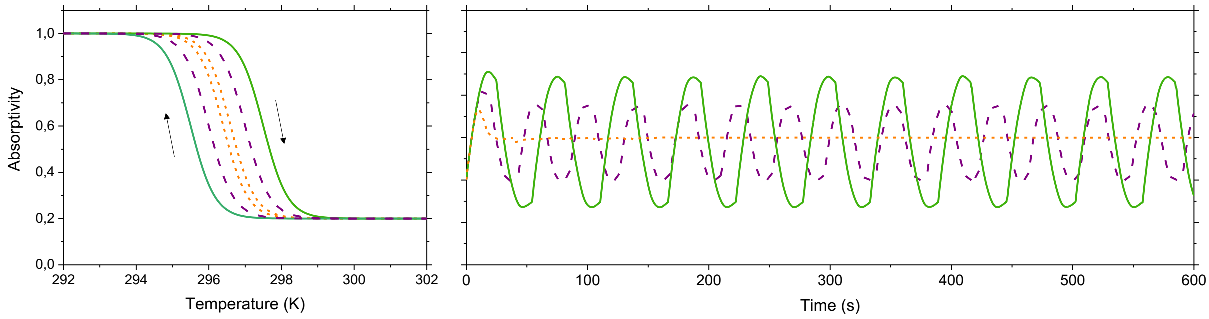


**Figure S22** Oscillatory response for hysteresis with different temperature threshold. Broader hysteresis results in a larger loop and a decrease of the frequency (no oscillation are observed for no hysteresis or very narrow hysteresis).

This simple analysis suggests that by varying the sorption properties and optical response of the porous media, different oscillatory behaviors can be achieved. This may further explain why heterogeneous packing of ZIF-8 on the antenna leads to variations in oscillatory behavior as discussed in Figure 5.

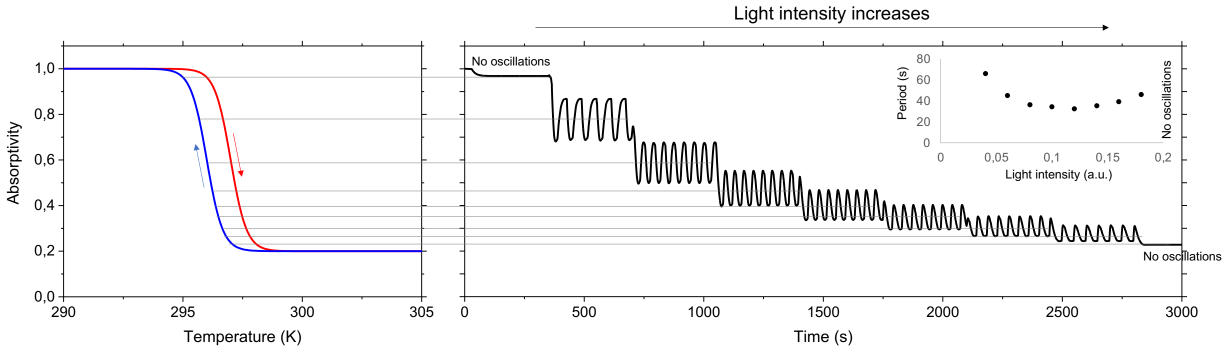


**Figure S23** Effect of light intensity. Simulations with increasing light intensity (0.02 increment every 400s). The oscillation period decreases with light intensity until the absorptivity reaches a low value, where the steepness of the hysteresis decreases, causing the oscillations to slow down and then stop.

| study | materials | mechanism | wavelenght of incident light | T ranges | optical change when heating | thermo-optical sensitivity |
|---|---|---|---|---|---|---|
| this work | plasmonic antenna / MOFs | light→heat→ plasmonic shift | 852 nm | 20 - 26.8°C | 0.6 decrease in absorptivity | 0,088 |
| Ref [13] | Liquid crystal elastormers | light→heat→ actuation | 488 nm | 22 - 49°C | 0.6 decrease in transmittance | 0,022 |
| Ref [14] | 1D photonic crystal / $RuO_2$ | light→heat→ photonic shift | 532 nm | 0 - 30°C | 0.6 decrease in transmittance | 0,020 |
| Ref [15] | $VO_2/SiO_2/Au$ | heat→ emission | - | 50 - 82°C | 0.24 increase in emissivity | 0,008 |
| Ref [16] | patterned $VO_2/SiO_2/Al$ | heat→ emission | - | 35 - 80°C | 0.48 increase in emissivity | 0,011 |
| Ref [17] | $VO_2$ antennas composite | heat→ emission | - | 50 - 65°C | 0.56 increase in emissivity | 0,037 |

**Figure S24** Comparison with different thermo-optical self-regulating systems

1. Dhakshinamoorthy, A., Navalón, S., Asiri, A. M. & Garcia, H. Gold-Nanoparticle-Decorated Metal-Organic Frameworks for Anticancer Therapy. *ChemMedChem* **15**, 2236–2256 (2020).
2. Muhamed, S., Aparna, R. K., Karmakar, A., Kundu, S. & Mandal, S. Immobilization of Gold Nanoparticles on Postsynthetically Modified NU-1000 for Hydrogen Evolution Reaction. *Inorg. Chem.* **62**, 7195–7202 (2023).
3. Huang, K. *et al.* Ultrathin ZIF-8 wrapping on Au-dotted Ag-nanowires for highly selective SERS-based CO2 gas detection. *Chem. Commun.* **57**, 2144–2147 (2021).
4. Liu, X. *et al.* Solar-Light-Driven Renewable Butanol Separation by Core–Shell Ag@ZIF-8 Nanowires. *Advanced Materials* **27**, 3273–3277 (2015).
5. Chen, Y.-T., Hsieh, N.-W. & Hsu, S.-W. Plasmonic Nanocrystal-MOF Nanocomposites as Highly Active Photocatalysts and Highly Sensitive Sensors for CO2 Reduction over a Wide Range of Solar Wavelengths. *Small Methods* **n/a**, 2500081 (2025).
6. Amyar, H. *et al.* In situ infrared spectroscopic ellipsometry as a tool to probe the formation of sol–gel based mesoporous films. *Journal of Sol-Gel Science and Technology* 1–12 (2023).
7. Dalstein, O. *et al.* Nanoimprinted, Submicrometric, MOF-Based 2D Photonic Structures: Toward Easy Selective Vapors Sensing by a Smartphone Camera. *Advanced Functional Materials* **26**, 81–90 (2016).
8. Johnson, P. B. & Christy, R.-Wjp. Optical constants of the noble metals. *Physical review B* **6**, 4370 (1972).
9. Chehadi, Z., Boissière, C., Chanéac, C. & Faustini, M. Nanoconfined water vapour as a probe to evaluate plasmonic heating. *Nanoscale* **12**, 13368–13376 (2020).
10. Wei, W., Huang, J., Li, W., Peng, H. & Li, S. Impacts of ethanol and water adsorptions on thermal conductivity of ZIF-8. *The Journal of Physical Chemistry C* **123**, 27369–27374 (2019).
11. Thorimbert, F., Odziomek, M., Chateau, D., Parola, S. & Faustini, M. Programming crack patterns with light in colloidal plasmonic films. *Nature Communications* **15**, 1156 (2024).

12. Thorimbert, F., Rivadeneira, M. C. & Faustini, M. Self-Assembling Cracks to Fabricate Antireflective Antifogging Metasurfaces. *Advanced Optical Materials* **13**, 2500079 (2025).
13. Zeng, H., Wani, O. M., Wasylczyk, P., Kaczmarek, R. & Priimagi, A. Self-Regulating Iris Based on Light-Actuated Liquid Crystal Elastomer. *Advanced Materials* **29**, 1701814 (2017).
14. Byun, C., Ceratti, D. R., Mimoso, C., Boissière, C. & Faustini, M. A Homeostatic Photonic Device Integrating Vapor-Regulated Thermo-Optical Feedback Mechanisms. *Advanced Functional Materials* **n/a**, 2424453 (2025).
15. Morsy, A. M. *et al.* Experimental demonstration of dynamic thermal regulation using vanadium dioxide thin films. *Scientific Reports* **10**, 13964 (2020).
16. Sun, K. *et al.* VO2 Thermochromic Metamaterial-Based Smart Optical Solar Reflector. *ACS Photonics* **5**, 2280–2286 (2018).
17. Ramirez-Cuevas, F. V. *et al.* Infrared thermochromic antenna composite for self-adaptive thermoregulation. *Nature Communications* **15**, 9109 (2024).
