# Supplementary material for: Self-regulating and self-oscillating metal-organic framework hybrid plasmonic metasurfaces
Source: Nat Commun. 2025 Nov 24;16:10392. doi: 10.1038/s41467-025-65338-2 (PMC12644829; doi:10.1038/s41467-025-65338-2)
Supplement: Supplementary file 3 — Description of Additional Supplementary Files [file 41467_2025_65338_MOESM3_ESM.pdf]

Description of Additional Supplementary Files

File name: Supplementary Video 1

Description: Evolution of the scattering intensity of the MOF hybrid plasmonic metasurface at increasing incident light intensity.

File name: Supplementary Video 2

Description: Animation and optical scattering evolution of the self-oscillating single antenna MOF composite.
